# Bridging the academic and practice/policy gap in public health: perspectives from Scotland and Canada

**DOI:** 10.1093/pubmed/fdy127

**Published:** 2018-07-24

**Authors:** J McAteer, E Di Ruggiero, A Fraser, J W Frank

**Affiliations:** 1 Scottish Collaboration for Public Health Research and Policy, School of Health in Social Science, University of Edinburgh, Teviot Place, Edinburgh, UK; 2 Dalla Lana School of Public Health, University of Toronto, Health Sciences Building, 155 College Street, Suite 408 Toronto, Ontario, Canada; 3 Public Health Science, NHS Health Scotland, Gyle Square, 1 South Gyle Crescent, Edinburghx, UK; 4 The Usher Institute, University of Edinburgh, Teviot Place, Edinburgh, UK

**Keywords:** organizations, public health, research

## Abstract

This article presents a critical commentary of specific organizational models and practices for bridging ‘the gap’ between public health research and policy and practice. The authors draw on personal experiences of such models in addition to the wider knowledge translation and exchange literature to reflect on their strengths and weaknesses as implemented in Scotland and Canada since the early 1990s.

## Background and rationale

The challenge of bringing health research findings to bear on relevant professional practices and public policies in areas such as public health is well documented.^1^ Prodigious growth has occurred within the ‘knowledge translation and exchange’ (KTE) field over the last 2 decades, starting in health services research,^2^ moving steadily through ‘evidence-based medicine’ driven by clinical research^3^ and more recently via an analogous thrust in population and public health research.^4^ Thus much is known about ‘what works’ to move research to action in these fields, and considerable implementation of those effective strategies has occurred. Despite this, ‘the gap’ still remains. In Canada the ground-breaking Naylor *et al.*,^5^ report on what happened (and did not, but should have) in the SARS outbreak of 2003, led to the creation of the Public Health Agency of Canada (PHAC) within a year (http://www.phac-aspc.gc.ca/). This report highlighted a lack of coordination among federal and other agencies in developing capacity to use evidence appropriately and a number of research priorities that were disconnected from the needs of public health practice. PHAC was partly created to help overcome this ‘gap’ by upgrading the research and research-utilization capacity of the public health policy and practice community. More widely, only around half of public health programmes and policies are reported as evidence based in the USA and the UK.^6,7^ Certainly, much work has been conducted recently in relation to this particular issue.^8,9^

This paper presents a critical commentary of specific organizational models and practices for facilitating collaborative partnerships between research, policy and practice in an effort to bridge the gap,^10^ drawing on the experiences of the authors in two countries, Scotland and Canada (The authors bring complementary expertise to this task: Frank and Di Ruggiero were the inaugural Scientific Director and Associate Director, respectively, of the CIHR’s Institute of Population and Public Health, from 2000 to 2008—a setting where improving the application of research to policy and practice was an explicit objective. McAteer and Frank have been involved in the establishment and operations of the Scottish Collaboration for Public Health Research and Policy, alongside Dr Ruth Jepson, at the University of Edinburgh, since 2008, a Centre expressly funded to improve linkage between public health research and its applied utilization in Scotland (www.scphrp.ac.uk). Di Ruggiero continued as deputy scientific director at the CIHR-IPPH until 2016 and is now with the University of Toronto’s Dalla Lana School of Public Health. Fraser, besides acting as the Chair of SCPHRP’s Advisory Council, made up of its stakeholders’ representatives, is also the Director of Public Health Science at NHS-Health Scotland, the nation’s major public health policy and programme think-tank).

## Factors contributing to the gap

Among the major reports about the public health research-to-action gap, the following underlying factors have been mentioned as contributing to both the origins of the gap, and its perpetuation:

- Context and complexity are pertinent factors to consider. Public health professionals are challenged by scope and scale (the health of populations versus the health of individuals), and the number of actors with whom they need to interact within and outside the health sector to facilitate change. Evidence-based medicine has been able to convince many practitioners, especially in teaching and academic settings, that better patient outcomes, at lower cost, can be achieved by more adherence to what high-quality studies have found.^11^ This process has not been as straightforward in public health—partly because of the difficulty of using conclusive RCT study designs to compare different interventions’ effectiveness—although significant methodological progress has recently been made in the design and analysis of non-RCT studies of population-level interventions.^12,13^
- Despite some improvements, public health continues to be characterized, by formal reviews on both sides of the Atlantic, as ‘silo'd’ in terms of the relationship between its research arm, based mostly in universities, and its practice and policy arm, based largely in public sector institutions such as Ministries and agencies. In the UK alone, major national reviews since 2001 of the ‘public health sector’ have criticized the tendency of academically oriented researchers in relevant disciplines to investigate and publish excessively theoretical and impractical studies of little use in policy and practice.^14–17^ The organizational structure of professional public health practice, in many high income countries (HIC), is strikingly removed from academia, unlike clinical research’s close ties to practice (at least within academic health science centres). Typically, public health professionals either report to local government (England and Wales since 2014 and before 1974, and much of English Canada and the USA for over a century) OR to a professional hierarchy often situated within a national health service (e.g. the 1974–2014 NHS public health arrangement in England and Wales, still in place in Scotland today, and arguably the entire US national Public Health Service). This separation takes many public health practitioners out of research-oriented settings, often situating them in governmental and other settings where research is only one of many influences on policy.^18^ Exacerbating this situation is the completely different reward structure for most academics, based mostly on publications, grants and trainee completions, compared to the more intra-organizational and professional reward system in public health practice and ‘policy shops’.
- The nature of many public health academic settings is more like the most traditional schools and faculties within higher education—rather unlinked to policy and practice. Again, this is quite unlike academic health science centres’ emphasis on ‘bench to bedside’ translation of clinical research, for better patient care. Thus it is not uncommon for full-time or ‘core’ faculty in public health-related university departments and Schools in Canada and the UK to have never practised public health professionally; to have no formal ties to such practice (in terms of their current academic job description—as opposed to being actively cross-appointed to the local ‘Public Health Department’); and to choose research topics which are typically uninfluenced by local practitioner or policy-maker opinion of what would be useful, or meet the needs of local decision-makers.
- This separation has been historically aided and abetted by research funding agencies, largely due to the practice of filling peer-review grant panels entirely with academics. Some progress has been made in certain research funder settings to incorporate the views of policy and practice ‘users’ of research in the prioritization of topics put forward through ‘Requests/Calls for Proposals’ (e.g. famously at Lomas’ Canadian Health Services Foundation since 1997) (now the Canadian Foundation for Healthcare Improvement: http://www.cfhi-fcass.ca/); at CIHR IPPH since 2000 (http://www.cihr-irsc.gc.ca/e/13777.html), and the NIHR Public Health Research Fund in the UK (https://www.nihr.ac.uk/funding-and-support/funding-for-research-studies/funding-programmes/public-health-research/).

The UK Research Excellence Framework (2014) recently placed greater emphasis on knowledge to action/‘impact’ in its methodology (admittedly still under development) for assessing, and economically rewarding, the top research institutions in the UK (http://www.hefce.ac.uk/rsrch/REFimpact/). However, it is still too early to judge the effects of that change on research productivity, subsequent societal impact (although some initial attempts have been made (http://blogs.lse.ac.uk/impactofsocialsciences/2017/07/19/what-do-the-2014-ref-results-tell-us-about-the-relationship-between-excellent-research-and-societal-impact/)) and researchers’ selection of topics investigated, in terms of links to policy and practice.

## Organizational models and innovative practices to overcome the gap

1) Centres explicitly charged with bridging the gap (through jointly produced research and knowledge mobilization to action):

Examples include:

- A national, public sector research funding agency with a strong corporate commitment to knowledge mobilization, such as the ‘CIHR Institute of Population and Public Health’ (IPPH).^19^ As the only public health-oriented CIHR Institute, out of thirteen created in 2000 when CIHR arose out of a major re-organization of the Medical Research Council of Canada, IPPH has for seventeen years been guided by the key principles of bridging the gap, as embodied in two practices: (i) its stakeholder-based approach to identifying priority topics for its many calls for research proposals and (ii) its evaluation of the policy and practice impacts of that research afterwards, including the uptake of research findings by decision-makers.^20^ More recently, the second wave of CIHR IPPH leadership has demonstrated that this approach is capable of building, within a decade, an entire applied field of public health research—intervention development, implementation and evaluation—which speaks to decision-makers’ need for research findings which can guide policy and practice more directly than has been the case in the past.^21–23^
- The six Canadian ‘National Collaborating Centres for Public Health’, funded by the Public Health Agency of Canada (PHAC) since the mid-2000s (https://www.canada.ca/en/public-health/services/public-health-practice/national-collaborating-centres-public-health.html). These Centres are a good example of non-academic ‘knowledge brokers’ to synthesize, interpret and disseminate research for policy and practice users.^24^ Notably, these Centres were not funded to actually do any research themselves, and some have been hosted in non-research institutions across Canada. Their history reveals both pros and cons of this model. In particular, they require a dedicated stream of funding, as has been provided federally by PHAC in this case, because neither universities oriented to research grant funding, pure research funding agencies, nor policy and practice organizations are likely to come up with the significant resources required for this sort of bridging activity. One challenge in their operation is to ensure that the Centres situated in non-research environments maintain close enough links with bona fide public health researchers to both utilize the most up-to-date methods for knowledge synthesis and dissemination, as well as maintain the respect of the national/global researcher community.
- The campus-based (but strongly community-partnered) ‘Scottish Collaboration for Public Health Research and Policy’ (www.scphrp.ac.uk). Since its founding in 2008, SCPHRP has utilized stakeholder consultations and partnering to: (i) identify major Scottish health problems; (ii) devise—jointly with community groups and NGOs, policy makers and public health professionals—novel programmes and policies to tackle them; and (iii) evaluate these interventions robustly, usually with mixed quantitative and qualitative methods.^25,26^ SCPHRP operates at national and community levels with policy makers, practitioners and researchers in pursuit of the shared goal of improving health in Scotland. The organization is committed to co-production; high academic and ethical standards; strong and effective communication; and to providing relevant topic and methodological expertise. Unusually, SCPHRP is funded by two traditional research funding agencies: the Medical Research Council of the UK, and the Scottish Chief Scientist Office; it is to their great credit that they joined forces in 2008 to fund SCPHRP, based on a visionary view of what was needed to move public health research towards better use in policy and practice. SCPHRP is a member of a larger group of five other Centres of Excellence in Public Health across the UK—also funded since 2008 by the MRC-led Clinical Research Consortium of diverse national research funders (http://www.ukcrc.org/research-coordination/joint-funding-initiatives/public-health-research/). They are similarly oriented to bridging the gap between research and policy/practice, across the UK.

It should be noted that good co-governance of evidence is fundamental to the success of such centres in meeting their aims and objectives.^27^

2) Provision of funding and incentives for meaningful cross-appointments:

- No such large-scale programme in Scotland or Canada are known to the authors from the recent period (which is in itself perhaps telling); there are elements of the NIHR Collaborations for Leadership in Applied Health Research and Care (CLAHRCs) across England which have strong cross-appointment features, in that academically appointed researchers are funded to work closely with local public health professionals to analyse practical problems in NHS services (both clinical and public health) and to find and evaluate solutions.^28^

3) On-the-job applied research training for public health professionals:

- The ‘SCPHRP Professional Part-Time PH Fellowship’ (2013–17), provided full-time NHS Public Health professionals with SCPHRP faculty mentoring on one research project each, agreed by Directors of Public Health across Scotland as corporate priorities, with the aim of strengthening the methodology of those projects, allowing them to be presented at public health professional/scientific conferences, and (ideally) published in an appropriate peer-reviewed journal. One such project involved a situation analysis exploring the views of health professionals working with women of childbearing age on current and future delivery of preconception care in an NHS board area in Scotland. This work has since influenced NHS board policy and practice (e.g. decision making related to preconception health) in addition to leading to publication in a peer-reviewed journal.^29^

4) Provision of honorary appointments for academics within public health bodies and vice-versa

- The Information Services Division (ISD) at NHS Scotland provides health information, health intelligence, statistical services and advice that supports the NHS and Scottish Government in public health matters. ISD operate a small-scale model of tethered academic work through Honorary Consultants arrangements. Similarly, SCPHRP have recently offered Visiting Expert positions within the University of Edinburgh to public health practitioners and decision-makers, with a view to facilitating links between research, policy and practice. These arrangements are typically small-scale, although are beneficial to both academic and non-academic partners.

5) Specific KTE strategies to enhance joint working by public health researchers and research users

- SCPHRP and NHS-Health Scotland have, with other local partner organizations, recently launched a novel ‘Public Health Intervention Evaluability Assessment Service’.^30^ It offers public and non-profit organizations a rigorous assessment of the evaluability of any public health programme or policy that is either already implemented or—ideally—being considered for implementation in the future. The methods used are well described in recent publications.^31^ This service does require resources from local ‘research brokering’ organizations, but has the potential ‘quid pro quo’ that researchers affiliated with those organizations can thereby obtain advance notice of potential opportunities to bid for subsequent evaluation contracts, or write grants for such work. There is therefore the potential for the service to substantially increase the volume of higher-quality evaluations completed in public health policy and practice settings.

## Conclusions and recommendations

Table 1 presents our views in relation to the strengths and weaknesses of each approach. Each of the above approaches to ‘bridging the gap’ between the two public health worlds—research versus policy and practice—has its strengths and weaknesses. Strikingly, published evaluations that have used strong scientific methods to assess such strengths and weaknesses are more limited, although there is some evidence that may be relevant especially for knowledge brokering.^32–35^ We recommend that those involved in any of the approaches described here to bridging the gap, or other novel approaches, invest in proper evaluation studies to learn precisely where, and why some of them do or do not achieve their potential. Ideally, we suggest that a given approach must be consistently implemented for at least a few years, in order to be able to realistically expect any impact on the gap—given its longstanding nature, and the many factors (see above) that perpetuate it.

**Table 1:** Perceptions of strengths and weaknesses in relation to approaches to bridging the gap

Approach	Strengths	Weaknesses
1) Centres explicitly charged with bridging the gap		
(i) Research funding agency	Allows research funding levers to be used to incentivize KTE activities among grantees	Can lead to ‘tick box’ KTE activities as grantees seek to be funded *per se*
(ii) Non-academic knowledge brokering centres	Fosters development of bespoke staff with research synthesis, communication and dissemination skills	Can isolate knowledge brokers from research expertise, leading to lower quality syntheses
(ii) Research centres with a mandate to broker	Integrates researchers who do projects into results’ synthesis, communication and dissemination	Can stretch Centres beyond normal academic roles: may not be institutionally rewarded
2) Provision of meaningful cross-appointments	Targets root problem: separate worlds of research versus policy/practice	Can stretch cross-appointees across ‘two masters’—conflicting performance criteria
3) On-the-job research training for PH professionals	Also targets root problem, by bringing research expertise into policy/practice settings	Very slow to achieve critical mass (such mentoring is labour-intensive); hard to fund
	Potential to develop such placements into jointly service/research funded posts	Does not directly tackle the barriers to promoting an evidence-based organizational culture
4) Provision of honorary appointments for academics within public health bodies and vice-versa	Targets root problem, by bringing research expertise into policy/practice settings and policy/practice expertise into research settings	Typically small-scale, and such appointment are often unpaid
5) Specific KTE strategies to increase joint working: e.g. programme/policy evaluability assessment services	Can target root problem, by bringing researchers and decision-makers together on joint projects, in a win-win situation. Can potentially lead to evaluation opportunities.	Potentially expensive for knowledge broker and applied research/KTE organizations to maintain if offered at no-cost

This short summary of our personal experiences, in the public health research and professional systems of Scotland and Canada, is intended to provoke further reflection from the Journal’s readers on the issue of how best to close the gap. We look forward to hearing those reflections.
